# Population Genomic Analysis of North American Eastern Wolves (*Canis lycaon*) Supports Their Conservation Priority Status

**DOI:** 10.3390/genes9120606

**Published:** 2018-12-04

**Authors:** Elizabeth Heppenheimer, Ryan J. Harrigan, Linda Y. Rutledge, Klaus-Peter Koepfli, Alexandra L. DeCandia, Kristin E. Brzeski, John F. Benson, Tyler Wheeldon, Brent R. Patterson, Roland Kays, Paul A. Hohenlohe, Bridgett M. von Holdt

**Affiliations:** 1 Department of Ecology & Evolutionary Biology, Princeton University, Princeton, NJ 08544, USA; eh7@princeton.edu (E.H.); lrutledge@trentu.ca (L.Y.R.); decandia@princeton.edu (A.L.D); kbrzeski@mtu.edu (K.E.B.); 2 Center for Tropical Research, Institute of the Environment and Sustainability, University of California, Los Angeles, CA 90095, USA; iluvsa@ucla.edu; 3 Biology Department, Trent University, Peterborough, ON K9L 1Z8, Canada; 4 Center for Species Survival, Smithsonian Conservation Biology Institute, National Zoological Park, Washington, DC 20008, USA; klauspeter.koepfli527@gmail.com; 5 Theodosius Dobzhansky Center for Genome Bioinformatics, Saint Petersburg State University, 199034 Saint Petersburg, Russia; 6 School of Forest Resources and Environmental Science, Michigan Technological University, Houghton, MI 49931, USA; 7 School of Natural Resources, University of Nebraska, Lincoln, NE 68583, USA; jbenson22@unl.edu; 8 Environmental & Life Sciences, Trent University, Peterborough, ON K9L 0G2, Canada; tyler.wheeldon@ontario.ca (T.W.); brent.patterson@ontario.ca (B.R.P.); 9 Ontario Ministry of Natural Resources and Forestry, Trent University, Peterborough, ON K9L 0G2, Canada; 10 North Carolina Museum of Natural Sciences and Department of Forestry and Environmental Resources, North Carolina State University, Raleigh, NC 27601, USA; rwkays@ncsu.edu; 11 Department of Biological Sciences, University of Idaho, Moscow, ID 83844, USA; hohenlohe@uidaho.edu

**Keywords:** canine, geographic isolation, spatial structure, restriction-site associated DNA, conservation genomics

## Abstract

The threatened eastern wolf is found predominantly in protected areas of central Ontario and has an evolutionary history obscured by interbreeding with coyotes and gray wolves, which challenges its conservation status and subsequent management. Here, we used a population genomics approach to uncover spatial patterns of variation in 281 canids in central Ontario and the Great Lakes region. This represents the first genome-wide single nucleotide polymorphism (SNP) dataset with substantial sample sizes of representative populations. Although they comprise their own genetic cluster, we found evidence of eastern wolf dispersal outside of the boundaries of protected areas, in that the frequency of eastern wolf genetic variation decreases with increasing distance from provincial parks. We detected eastern wolf alleles in admixed coyotes along the northeastern regions of Lake Huron and Lake Ontario. Our analyses confirm the unique genomic composition of eastern wolves, which are mostly restricted to small fragmented patches of protected habitat in central Ontario. We hope this work will encourage an innovative discussion regarding a plan for managed introgression, which could conserve eastern wolf genetic material in any genome regardless of their potential mosaic ancestry composition and the habitats that promote them.

## 1. Introduction

Neutral theory provides a strong foundation for the application of conservation genetics, which identifies evolutionary distinct lineages that are vulnerable to genetic erosion or extinction due to reductions in population sizes [1,2,3]. Historically, conservation studies have had to rely on a small number of neutral loci for genetic surveillance, even though the consequences of reduced effective population sizes may manifest genome-wide (e.g., [4]). The advent of modern genomic methods has allowed the surveys of putatively neutral, unlinked loci distributed across the genome, enabling higher confidence in genetic guidance of conservation efforts and policy, for example, when facing decisions about isolated populations and the accompanying loss of genetic diversity.

Island species have been used as archetypal evolutionary models representing the consequences of isolated populations that can persist despite low effective population sizes [5,6,7,8]. However, these examples represent the simplest of scenarios, where closely related, potentially hybridizing congeners are rarely a factor contributing to loss of genomic diversity. For many mainland species, genetic drift in small populations may be only one of many evolutionary processes that threaten intraspecific diversity; hybridization and introgression may represent an equal or greater threat to parental species’ genomic integrity. As individuals disperse from isolated populations, they likely experience low densities of conspecifics, and have an increasing probability of interbreeding with nearby congeners. If there is a lack of reproductive isolating barriers, fertile admixed offspring can be produced (e.g., [9,10,11,12]). Assuming the isolated population is of management concern (i.e., threatened/endangered species), the consequences of natural source-sink dynamics can also result in challenges for species protection regulations. Admixed individuals continue to carry fractions of their genome derived from the endangered or managed parental species. However, the U.S. Endangered Species Act of 1973 lacks any specific guidelines or regulation regarding admixed or hybrid individuals [13,14,15]. The Committee on the Status of Endangered Wildlife in Canada (COSEWIC) does clarify that conservation decisions should consider the consequences of hybridization, particularly anthropogenic hybrids that threaten the status of a parental species [14,16]. Further, if hybrids actively supplement the genetic diversity of a depauperate population under conservation consideration, COSEWIC supports hybrid conservation [14,16].

Two scenarios will accelerate deterioration of the hybrid genome representing the rare parental species. First, extensive backcrossing with the more common parental species will result in rapid loss of the divergent biodiversity found in the rarer parental species (e.g., [17,18,19,20]). Second, genome exclusion can increase extinction risk if there is a lack of recombination between parental genomic fragments, resulting in the lack of representation of one parental genome in each subsequent admixed offspring (e.g., [21,22,23]). An example of genomic replacement has been documented in a newly discovered species of canid, the African wolf, *Canis anthus* [24], recently reclassified as *C. lupaster* [25]. Consequently, there is a call for conservation concern for the newly described and unique evolutionary legacy of *C. lupaster*. This example illustrates the potential for misaligned policy if conservation units have been established predominantly from mitochondrial DNA (mtDNA), a single linkage group that represents the matriline that is difficult to interpret for interbreeding lineages with incomplete lineage sorting (e.g., [26,27,28]).

The eastern wolf (*C. lycaon*) is an endangered canid with an evolutionary history obscured by recent hybridization with its North American congeners, the gray wolf (*C. lupus*) and recently expanded coyote (*C. latrans*) (e.g., [29,30,31,32,33,34,35,36]). The genetic distinction of eastern wolves has been reported with nuclear microsatellites in combination with maternal (mtDNA) and paternal (Y-chromosome) markers [29,37]. Although conclusions from genomic analyses have been contradictory with respect to their evolutionary origins (e.g., [33,34,36]) there has been consensus regarding the importance of eastern wolf conservation. Previous microsatellite surveys revealed eastern wolves were predominantly found in several protected areas in central Ontario: Algonquin Provincial Park (APP; 7653 km^2^), Queen Elizabeth II Wildlands Provincial Park (QEWPP; 225 km^2^), Killarney Provincial Park (KPP; 485 km^2^), and Kawartha Highlands Provincial Park (KHPP; 376 km^2^) [37,38,39]. Eastern wolves have also been found in unprotected areas in central Ontario [38,39], although their occurrence outside protected areas is relatively rare where they experience high rates of human-caused mortality [40]. Numerical and geographical expansion of eastern wolves outside of APP and the smaller protected areas appears to be limited by human-caused mortality, hybridization, territoriality with other canids, and interactions between these processes [39,40,41]. Both COSEWIC and the Committee on the Status of Species at Risk in Ontario (COSSARO) have policies regarding genetic admixture and have formally recognized the eastern wolf as a listable entity, with the latter recognizing admixture from gray wolves and coyotes [14,16,42,43]. Collectively, past research has repeatedly raised the question of conservation priorities, especially if the eastern wolf genome is geographically restricted.

Given the potential for widespread hybridization, introgression, and dispersal across the region, we evaluated contemporary population genetic dynamics and conservation value to expand on the study of Rutledge et al. [35] by using a similar genomic approach across a broader representation of canids. Our objective was to uncover spatial patterns of genetic variation in canids across central Ontario, Canada. We further tested the identity of samples obtained by a furbearer organization in central Ontario to address the concern of species identification through non-genomic assessments. This molecular approach enabled us to investigate genomic variation across a dense, regional representation of over 300 North American canids for exploring landscape-level dynamics.

## 2. Materials and Methods

### 2.1. Sample Collection and DNA Extraction

**Figure 1:**
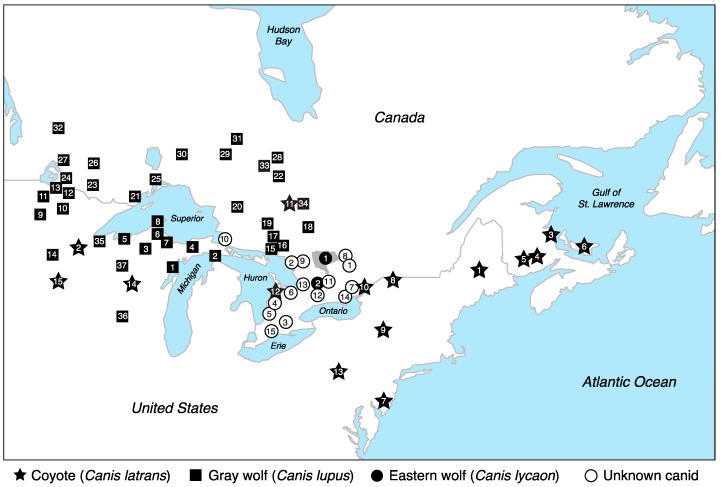
Map of the Great Lakes and central Ontario regions of North America with sample locations of three *Canis* species (site number indicated in symbol) for 30 eastern wolves, 96 gray wolves, 127 coyotes, and 51 unknown canids. Eastern wolf site 1 is Algonquin Provincial Park (gray shaded region); eastern wolf site 2 is Queen Elizabeth II Wildlands Provincial Park. See Table S1 for details on each sample’s geographic location.

We obtained blood, tissue, and DNA samples from 304 canids across the northeastern U.S. and central Ontario (Figure 1) that specifically included 127 eastern coyotes and 96 Great Lakes type gray wolves, which differ from their western counterparts in that they both have been impacted by hybridization [30,32,34,44,45,46]. Great Lakes type gray wolves are distributed throughout much of the Great Lakes states, Manitoba, Ontario, and Quebec [29]. Eastern coyotes are now widely distributed throughout much of Ontario, Quebec, the eastern provinces in Canada, and across the northeastern United States [32]. For this study, we hereafter refer to these *Canis* types as gray wolves and coyotes, respectively. We also obtained samples from 30 eastern wolves and included tissue samples from 51 canids of unknown taxonomic affiliation from central Ontario to increase sampling density around the focal geographic region surrounding APP. Samples were collected through state management programs, government/state organizations (e.g., US Department of Agriculture, Department of Natural Resources, Ontario Ministry of Natural Resources and Forestry, furbearers), or museum archives (New York State Museum). In all cases, sample origin was known at the state or provincial level and was associated with either a specific GPS location from the exact collection location or the township nearest the collection site. We extracted high molecular weight genomic DNA with the DNeasy Blood and Tissue Kit (Qiagen, Maryland, USA) or the BioSprint 96 DNA Blood Kit in conjunction with a KingFisher Flex Purification platform (Thermo Fisher Scientific, Waltham, MA, USA) following manufacturers’ protocols. We identified high-quality DNA as a high molecular weight band (>1 Kb) on a 2% agarose gel with a 2-log DNA ladder (New England Biolabs, Ipswich, MA, USA), quantified using either PicoGreen or Qubit 2.0 fluorometry (Thermo Fisher Scientific, Waltham, MA, USA), and standardized to a concentration of 5 ng/µL.

### 2.2. Reduced Representation Sequencing and Data Processing

We followed the modified restriction site associated DNA sequencing (RADseq) protocol [47] to prepare genomic libraries for 30 eastern wolves, 96 gray wolves, 127 coyotes, and 51 canids of unknown taxonomic affiliation from central Ontario (Figure 1, Table S1). Further, we constructed a second dataset of 317 canids, which included the original 304 wild canids with the addition of 13 domestic dogs to ensure that genetic structuring is not driven largely by admixture or hybridization with domestic dogs (for all details pertaining to this dataset, see Appendix).

Genomic DNA samples were digested with *sbfI* followed by ligation of a unique 8 bp-barcoded biotinylated adapter. We pooled 96–153 samples, which were then randomly sheared to 400 bp on a Covaris LE220. We enriched for adapter ligated fragments using a Dynabeads M-280 streptavidin bead binding assay (Thermo Fisher Scientific). We then prepared these final genomic libraries using either the NEBnext Ultra DNA Library Prep Kit or the NEBnext UltraII DNA Library Prep Kit as per the manufacturer’s instructions (New England Biolabs, Ipswich, MA, USA). We selected for genomic fragments between 300–400 bp in size using Agencourt AMPure XP magnetic beads. We then standardized libraries to 10nM, which were then paired-end sequenced (2 × 150 nt) on two lanes of the Illumina HiSeq 2500 at Princeton University’s Lewis-Sigler Institute for Integrative Genomics core facility. Using a custom perl script, we aligned both the forward and reverse raw sequencing reads to retain the read that contained the *sbfI* cut site along with a barcode and discarded all other reads.

We demultiplexed reads using the *process_radtags* function and a 2 bp mismatch in *STACKS* v1.42 [48]. With a sliding window approach (with a step size of 15% of the read length), we discarded reads that contained either uncalled bases or had low-quality scores (*Q* < 10). We subsequently removed PCR duplicates using the paired end filtering option with the *clone*_*filter* function. Samples with a minimum of 500,000 reads were retained and mapped using *STAMPY* v1.0.20 [49] with default parameters to the reference dog genome (CanFam3.1) [50]. Sites with low mapping quality (MAPQ < 96) were removed. We used *Samtools* V. 0.1.18 [51] to convert files to BAM format and discovered single nucleotide polymorphism (SNP) variants in *STACKS* following the recommended pipeline for data mapped to a reference genome (i.e., *pstacks* → *cstacks* → *sstacks* → *populations*), with the –m 3 flag to identify stacks that had a minimum of 3-fold coverage. A repeated analysis with a 10-fold coverage filter resulted in identical findings (data not shown). We executed the *populations* module twice to optimize the final sample selection, which works to reduce both missing data and biases resulting from uneven sampling across geographic locations and genetic groups. We reported only the first SNP per locus (--write_single_snp) and did not apply any missing data thresholds. We used *PLINK* [52] to calculate the total missingness per individual and removed individuals with >85% missing data. In the second execution of *populations*, we only reported loci that were genotyped in 90% of individuals (-r 0.9) and again restricted our analysis to only the first SNP per locus.

We estimated observed (H_o_) and expected (H_e_) heterozygosity, genetic differentiation (F_ST_), and the number of private alleles per evolutionary lineage within and between each evolutionary lineage and sampling location using *populations* in *STACKS*. We estimated private allelic richness in *ADZE* v. 1.0 with missing data tolerance set to 25% [53].

### 2.3. Clustering and Genetic Structure Analysis

To obtain a statistically unlinked set of SNP loci, we filtered for linkage disequilibrium (LD) in *PLINK* using the flag and parameters --indep-pairwise 50 5 0.5. We then filtered SNPs to retain those in Hardy–Weinberg Equilibrium (HWE) in *PLINK* (--hardy; *p* > 0.05). We additionally filtered to retain sites with a minor allele frequency of 1%. The expectation is that this SNP set contains putatively neutral loci useful for population genetic and demographic analyses, hereafter referred to as the “neutral SNP set”.

We visualized clustering using a principal component analysis (PCA) with the program *flashPCA* [54]. We next evaluated genetic structure using a maximum likelihood approach implemented in *ADMIXTURE* v1.3 with the cross-validation flag for *K* = 2–10 [55]. We analyzed both the full SNP and neutral SNP sets in *ADMIXTURE*, although such methods often assume the use of neutral markers, random mating, and a lack of immigration, with no violations in this subset of loci and population. The goal was to balance the signal between a full data set, which includes loci potentially under selection, and the identified neutral SNP set.

To conduct population assignment testing, we used two methods. The first test was a discriminant analysis of principal components (DAPC) completed with the function *dapc* in R’s *adegenet* library [56]. This multivariate approach uses a sequential *K*-means clustering method on SNP genotypes transformed using their principal components to conduct a discriminant analysis without violation. Although PCA is a computationally rapid tool to identify underlying genetic structure in a large data set, it only summarizes patterns in the data and lacks formal assessment methods. DAPC maximizes the model that explains between-group variation while minimizing that found within groups, and identifies the best fit number of genetic clusters. Additionally, we used discriminant analysis to probabilistically assign individuals to each genetic cluster identified.

We next obtained posterior probability population assignments for the 51 canids of unknown taxonomic affiliation in a Bayesian approach using the neutral SNP set in *STRUCTURE* [57,58,59,60]. We used the parameters *USEPOPINFO* = 1, *PFROMPOPFLAGONLY* = 1, *MIGPRIOR* = 0.01, and *GENSBACK* = 2 with 5000 burnins and 10,000 reps. We classified individuals as “reference” if they had high assignments (*Q* ≥ 0.9) to their corresponding clusters from the *ADMIXTURE* analysis. 

Finally, we constructed an unrooted neighbor-joining (NJ) tree [61] based on pairwise nucleotide distances to estimate the clustering among all North American canids using the neutral SNP set. The NJ tree was estimated in PAUP* v4.0a159 [62] with ties broken randomly and no topological constraints defined during the tree search. We also constructed an unrooted maximum likelihood tree with the Kimura 2-parameter model using W-IQ-TREE [63,64]. The topology from this analysis was largely concordant with that from the NJ analyses, and therefore, we only report the results from the latter analysis. The final output tree file (.tre) was saved and visualized using *FigTree* v1.4.3 [65].

We used *TreeMix* v1.13 [66] to infer population relationships, allowing up to 10 migration events (-m 0 to 10) across the neutral SNP set. Samples were grouped by region for analysis (see Table S1 for results of group membership).

### 2.4. Spatially Explicit Bayesian Clustering

We implemented two spatially explicit models in *Geneland* within the R framework [67]. We applied a stricter minor allele frequency filter of 3% to the neutral SNP dataset and retained a total of 985 SNPs for this analysis. For all runs, we converted latitude and longitude of sample locations (recorded in decimal degrees) to a planar coordinate system (UTM, Zone 17N) using the R package *PBSmapping* [68] to avoid distortion resulting from spherical coordinates, which is magnified as one approaches the poles. For each run, we used default settings in *Geneland*, except for the following: number of populations tested (Min = 1, Max = 8), maximum number of nucleotides (n = 985), number of iterations (1,000,000), and thinning (100). We ran multiple independent runs for each dataset and checked for convergence.

For each dataset used as input for *Geneland* (see below), we ran both an uncorrelated and a correlated allele [69] frequency model, to build spatially-explicit demographic structure maps that incorporate genomic structure, geography, and potential admixture. The uncorrelated allele frequency model assumes each population’s allele frequencies are completely independent of other population allele frequencies, and as such likely estimates a conservative probabilistic assignment of populations. The correlated allele frequency model assumes that allele frequencies in populations tend to be similar across populations, and may therefore detect more subtle differences between them (for example, small frequency differences in a rare allele in two populations will affect probability assignments greater using a correlated model). These models are typically seen as more speculative than the uncorrelated models [67].

We ran each of these models on two datasets: (1) all 281 canids and (2) a subset of 122 samples that included 36 coyotes, 30 eastern wolves, 13 gray wolves, and 43 canids of unknown taxonomic affiliation from geographic locations adjacent to and surrounding APP (Table S1). We processed and visualized the results of these models in *QGIS* [70] as follows. For each group *K_i_* identified from the total *K_t_* (selected by choosing the one with the highest estimated density of all clusters tested), we selected a color representing that group and calculated the mean probability of membership of *K_i_*. For all pixels identified as below the mean probability of membership for *K_i_*, we visualized these pixels as transparent (no color). For all pixels identified as above the mean probability of membership for *K_i_*, we assigned these pixels the corresponding color, with an intensity weighted by the actual probability score. In the final map, all pixels with values above the mean probability of membership for any *K_i_* will be colored with some color value, and all the pixels that did not score at least the mean probability of membership for any *K_i_* will not be colored.

## 3. Results

### 3.1. Eastern Wolves Are a Genetically Isolated Population of Wolves

We identified a total of 16,587 SNP loci in 304 canids from the Great Lakes region and central Ontario. The proportion of genotype missingness was moderate across all samples (average = 0.07, sd = 0.09, max = 0.45, median = 0.03, mode = 0.00) (Table S1). We found only 23 individuals had significantly high missingness (i.e., ≥92nd percentile of the missingness distribution or missingness ≥ 0.25): 13 coyotes (all sampled from Pennsylvania) and 10 gray wolves (Great Lakes region n = 5, Ontario n = 5). These 23 individuals were removed from all subsequent analyses of the remaining 281 canids (coyote n = 114, eastern wolf n = 30, gray wolf n = 86, unknown canids n = 51). We then filtered the SNP set to retain sites with a minor allele frequency of 1%, resulting in 5665 SNPs. Additionally, we filtered further to retain 3067 unlinked loci in HWE, representing the neutral SNP set.

Eastern wolves had significantly lower estimates of expected heterozygosity in both SNP sets compared to gray wolves and coyotes (1-tailed *t*-test of unequal variance *p* < 0.01) (Table S2). We noted the highest level of genetic differentiation was found between coyotes and gray wolves in the 5K SNP set (F_ST_: coyote-gray wolf = 0.029, gray wolf-eastern wolf = 0.022, coyote-eastern wolf = 0.021), with a similar trend noted for the neutral SNP set (F_ST_: coyote-gray wolf = 0.018, gray wolf-eastern wolf = 0.016, coyote-eastern wolf = 0.015). We found that across the 5K SNP set, eastern wolves displayed the fewest number of private alleles and lowest allelic richness (n, private alleles: eastern wolf = 12, gray wolf = 395, coyote = 1157; prop. richness: eastern wolf = 1.49, gray wolf = 1.54, coyote = 1.65) (Table S3A), with eastern and gray wolves comparable in their allelic diversity after adjusting for sample size differences (Figure S1).

**Figure 2:**
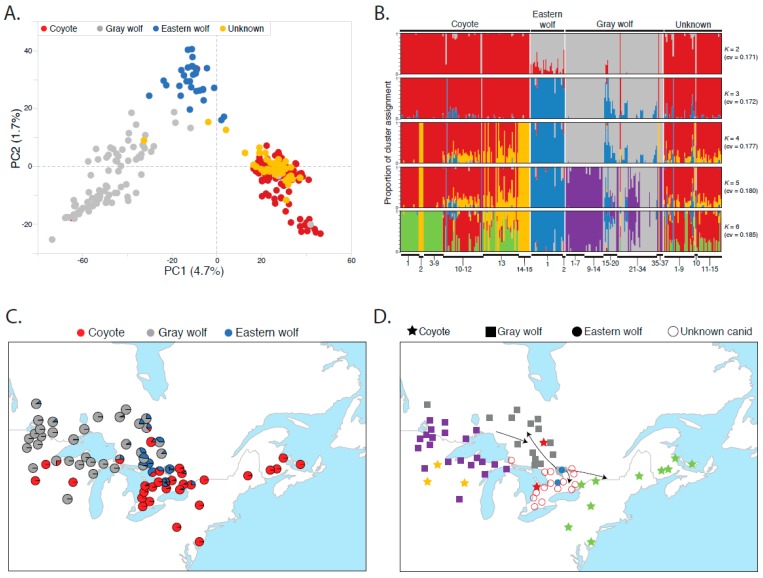
Genetic analysis of the 3067 neutral single nucleotide polymorphism (SNPs) genotyped in 281 canids (30 eastern wolves, 86 gray wolves, 114 coyotes, and 51 canids of unknown taxonomic affiliation) using (**A**) principal components analysis for clustering and (**B**) maximum-likelihood cluster membership with cross-validation (*cv*) values and site numbers from Figure 1 provided along the X-axis. (**C**) Visualization of per-site average cluster membership to each genetic group at *K* = 3 (site details, see Table S1). (**D**) A summary of the migration events that explicitly involve eastern wolves, as inferred by *TreeMix* from 3067 neutral SNPs genotyped in 281 canids. Arrows indicate the direction of inferred migration events. Color of symbols are consistent with genetic cluster membership from *K* = 6 in part (**B**), designating coyotes from the Great Lakes (yellow), Northeastern U.S. (green), and Ontario (red); eastern wolves (blue), gray wolves predominantly from the Great Lakes (purple) and Ontario (gray), and the unknown canids from central Ontario (red outline).

A principal component analysis (PCA) of all 281 canids reveals substantial genetic distinction of each species, with PC1 explaining 4.7% of the variation, while PC2 (1.7% of variation) separates eastern wolves from coyotes and gray wolves (Figure 2A and Figure S2). In the genetic structure analysis of the neutral SNP set, we found negligible differences in cross-validation estimates between the two- and three-cluster analyses (Figure 2B and Figure S3). We selected the *K* = 3 level of partitioning, which represents the genetic groups of coyotes, eastern wolves, and gray wolves with high assignments to their respective clusters (average *Q*_coyote_ = 0.95, *Q*_eastern wolf_ = 0.96, *Q*_gray wolf_ = 0.88 (Figure 2B). However, there was noticeable substructure within all groups at higher levels of partitioning, with samples of different species from different geographic regions distinctly clustering. For example, a fourth partition separated eastern coyotes (sites 1, 3–9, 10–12) from Great Lakes coyotes (sites 2, 14–15) (Figure 2B). Further, we observed shared assignments between eastern wolves and gray wolves (sites 15–22; average *Q* to eastern wolf cluster = 0.32) and coyotes (sites 10–12; average *Q* to eastern wolf cluster = 0.10) of central Ontario (Table S1). Averages of *Q* values at *K* = 3 for each geographic site revealed the spatial distribution of eastern wolf cluster membership proportions (Figure 2C). Average *Q* values across geographic sites suggest that there is a reduced yet detectable presence of eastern wolf genetic material in gray wolves and coyotes of central Ontario, with the highest concentration of eastern wolf alleles found within the canids sampled in APP and QEWPP (Figure 2C and Figure S4). We further included 13 domestic dogs in a genetic structure analysis and found that their inclusion did not explain the genetic clustering of eastern wolves, which carry minor proportions of dog ancestry at best although we encourage caution for interpretation of this result due to a small representation of dogs (check Supplementary).

We constructed a neighbor-joining tree from pairwise genetic distances among 281 canids and found that coyotes and gray wolves broadly formed two distinct clusters, with eastern wolves representing a group within the gray wolf cluster (Figure S5). We found low levels of discordant clustering: two of the 114 coyotes clustered with gray wolves, one coyote clustered with eastern wolves, three of the 86 gray wolves grouped with coyotes, eight gray wolves grouped with eastern wolves, and two of the 30 eastern wolves clustered with the gray wolves. We found that 11 individuals with discordant cluster memberships in the neighbor-joining tree also had admixed assignments at the most likely *ADMIXTURE* partition (*K* = 3, *Q* < 0.9, n coyote = 3; eastern wolves = 2; gray wolves = 6) (Figure S5; Table S1). The eastern wolves were nested within the gray wolf group, with only two individuals clustering outside of this group. Individual ID #2097 and #2100 clustered at the base of the gray wolf group in a small cluster with a coyote #6023 and an unknown canid #8633, both from the Frontenac Axis along the eastern border of Ontario adjacent to northern New York state (Figure S5). The caution for interpretation is that the neighbor-joining tree does not include an inherent Bayesian approach to fulfill Hardy-Weinberg expectations within populations. Overall, we found support for eastern wolves constituting their own discrete genotypic cluster.

We also used a discriminant analysis of principal components to assess population assignment. We retained 300 PCs that explained most of the cumulative variance that identified three genetic clusters based on the lowest Bayesian information criterion, and we selected to retain two linear discriminant functions for the analysis (Figure S6). We found further support of three genetic clusters while controlling for intra-group variation, with high assignments of individuals to their original assumed population (Figure S6, Table S1). However, seven individuals were not assigned to their respective known taxonomic group: two coyotes and five gray wolves (Table S1). Coyote individual #6017 was exclusively assigned to the eastern wolf genetic group and coyote individual #2311 was exclusively assigned to the gray wolf cluster in both *ADMIXTURE* and *dapc* analysis. Of the five discordantly grouping gray wolves, two were assigned at very high probabilities to the coyote group (individuals #7294 and #2338) while three were assigned to the eastern wolf group (individuals #7413, #7415, and #7344) (Table S1).

To determine if the presence of admixed individuals in the dataset influenced our assessment of allelic richness and private allele identification, we identified a subset of 186 individuals with high assignments at *K* = 3 to their respective clusters (*Q* > 0.9 n: coyote = 99, eastern wolf = 25, gray wolf = 62) (Table S1). We found patterns similar as before with respect to the number and richness of private alleles among the three species (n, private alleles: eastern wolf = 62, gray wolf = 574, coyote = 1718; prop. richness: eastern wolf = 1.43, gray wolf = 1.45, coyote = 1.59) (Table S3B).

### 3.2. Canids of Unknown Taxonomic Affiliation Carry Predominantly Coyote Genetic Variation

We genotyped 51 canids of unknown taxonomic affiliation that were archived by a furbearer organization in central Ontario. Due to a large range of morphologic variation of sympatric eastern coyotes, eastern wolves and Great Lakes type gray wolves (e.g., [37,38,71]), the taxonomic membership of these specimens was not assumed for this study. Of the 51 canids of unknown taxonomic affiliation, 49 of them were sampled in central Ontario proximal to APP, with only two individuals originating along the western shores of Lake Huron (site 10) (Figure 1). Here, we explored their genetic similarities to coyotes, gray wolves, and eastern wolves. We found that coyotes were the least differentiated from the unknown canids relative to the other canid groups (F_ST_: unknown canids to coyote = 0.001, unknown canids to eastern wolf = 0.005, unknown canids to gray wolf = 0.008), which is a result that was also supported by the neutral SNP set (F_ST_: unknown canids to coyote = 0.000, unknown canids to eastern wolf = 0.003, unknown canids to gray wolf = 0.005).

The neighbor-joining tree from pairwise genetic distances revealed that 46 of the 51 unknown canids grouped with coyotes, while two grouped within the eastern wolf cluster and three with gray wolves (Figure S5). We further assigned exclusive membership of these 51 unknown canids to genetic clusters using a discriminant analysis of principal components. We found 48 assigned to the coyote cluster, two assigned to the eastern wolf cluster, and a single canid assigned to gray wolf (Table S1, Figure S7).

Through an *ADMIXTURE* analysis, we found high assignments to the coyote cluster at *K* = 3 (average *Q*: coyote = 0.90 ± 0.22, eastern wolf = 0.09 ± 0.18, gray wolf = 0.01 ± 0.08) (Figure 2D, Table S1). Assignment to the eastern wolf cluster, however, was significantly higher than that to the gray wolf cluster (1-tailed *t*-test of unequal variance *p* = 0.0036), likely due to two individuals with substantial eastern wolf assignments: individual #8647 from geographic site 2 was nearly exclusively assigned to the eastern wolf cluster (*Q* = 0.999), while a second individual from site 9 shared assignments to both the eastern wolf and coyote cluster (#8629 *Q* = 0.604 and 0.396, respectively) (Figure 1, Table S1). Further, individual #8604 from site 10 had partial assignments to both eastern and gray wolf clusters (*Q* = 0.432 and 0.569, respectively). Assignments to the coyote genetic cluster remained high across all levels of partitioning, with partial assignments to coyotes from sites 1–9 and 13, likely indicative of allele sharing with coyotes from northeastern United States (Figure 2B). Using the 186 reference individuals identified due to high cluster membership, we used a Bayesian posterior probability assignment test (at *K* = 3) to obtain a statistical taxonomic assignment of these 51 canids. Assignments were high to the coyote cluster across all individuals, supporting the inference that these canids carry genetic variation exclusively found among coyotes (average posterior probability *Prob*(*Q*) ± sd: coyote = 0.91 ± 0.03, eastern wolf = 0.06 ± 0.02, gray wolf = 0.03 ± 0.02) (Table S4).

### 3.3. Eastern Wolf Genetic Variation Is Geographically Surrounded by Coyote Populations

We evaluated migration events with a graph-based model in *TreeMix v1.13*, which infers population splitting and mixing from genome-wide allele frequency data [66]. With the neutral SNP set, we surveyed gene sharing events across 281 canids (n coyote = 114, eastern wolf = 30, gray wolf = 86, unknown canids = 51) and found that the amount of variance explained increased with increasing migration events, with eight migration events maximizing the likelihood value (Figure S8; Table S5). We find that eastern wolves are grouped closest to gray wolves in eight of 11 graphs (*m* = 0, 1, 2, 3, 6, 7, 8, 10 in Figure S8), also supported by the neighbor-joining tree (Figure S5), suggestive of the genetic support that eastern wolves more closely align with gray wolves. The three discordant graphs show a placement of eastern wolves as basal to all groups (*m* = 4), aligned with coyotes of northern Ontario (*m* = 5), or basal to all coyotes (*m* = 9). However, we also noted a general trend that as more migration events are incorporated, a larger fraction of them were at higher weights. Thus, models with fewer migration events likely represented a better-fit due to the lower weights of the migration branches. When considering only migration events that involved eastern wolves, we found a dynamic pattern that was repeated across analyses: a flow of eastern wolf genomes to coyotes of central Ontario, with allele movement into gray wolves of northeastern Ontario (Figure 2D) The more striking result was the inferred movement of eastern wolf alleles from APP eastward towards coyotes of the Frontenac Axis in central Ontario near the New York state line (Figure 2D). These basic trends remained with increased migration events, only adding movements between various gray wolf and coyote populations.

### 3.4. Eastern Wolves Are Spatially and Genetically Isolated

**Figure 3:**
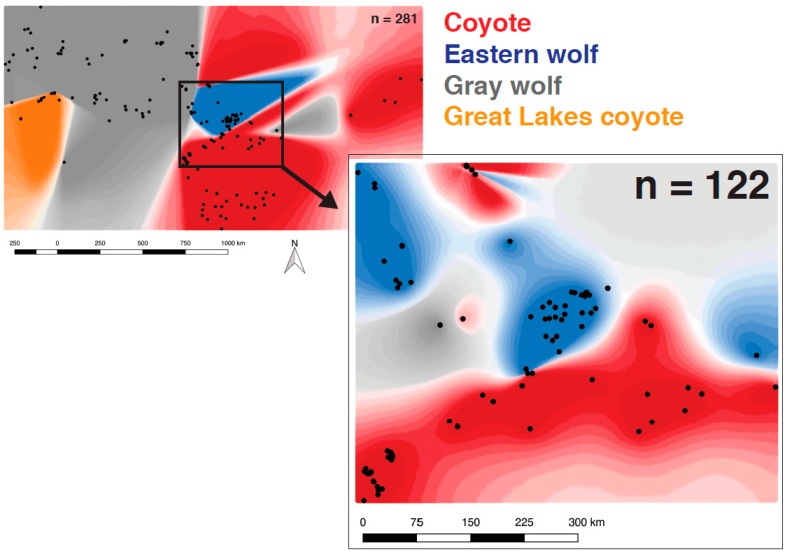
A spatially explicit analysis of the best supported partitions with a correlated-allele model using genotypes from 985 SNPs across 281 canids (upper left panel: red, eastern coyotes; blue, eastern wolves; gray, gray wolves; orange, Great Lakes coyotes). A geographically restricted analysis of 122 canids (lower right panel) identified three spatial and genetic groups as the most supported (see Table S1 for individual population assignment probabilities).

We built spatially explicit maps of genomic population structure in *Geneland* using 985 neutral SNPs that had a minor allele frequency of ≥3% across the full set of canids. Analysis of 281 canids of known taxonomy revealed two spatial and genomic clusters that split coyotes and eastern + gray wolves using the uncorrelated allele frequency model (maximum *a posteriori* estimate of *K* = 2, results not shown). The correlated allele frequency model revealed four distinct spatial and genomic clusters of canids, corresponding to two distinct genetic signatures of eastern and Great Lakes coyotes, a group of distinct eastern wolves, and a group of gray wolves (Figure 3, Table S1) and were concordant with structuring detected by *ADMIXTURE*. Of note, 44 of the 51 unknown canids grouped with coyotes, two with gray wolves, and five with eastern wolves.

We then geographically restricted the analysis to remove any signal from subdivisions and focused the investigation to APP and the surrounding region. When we analyzed 122 canids that originated from within and surrounding APP, we found that the maximum posterior probability from the correlated model supported three separate spatial and genomic clusters (uncorrelated model, *K* = 1) (Figure 3, Table S1). A single spatial genetic group contained 35 of the 36 coyotes and 41 of the 43 unknown canids analyzed. A second spatial genetic group contained all 30 eastern wolves and 13 gray wolves, as well as a single coyote (#2311). The remaining two unknown canids composed their own spatial group (ID #8645 and #8647). Although both have previous admixed signals, they were both assigned as coyote in a Bayesian posterior probability assignment test (Table S4).

Due to the smaller sample set included in this geographically restricted analysis (n = 122), there were sharp distinctions in spatial genetics noted along the southern geographic boundary of APP. We also observed a more western expansion of the eastern wolf spatial genetic group as gray wolf populations are noted to exhibit non-negligible amounts of allele sharing with eastern wolves (Figure 2C).

## 4. Discussion

For many endangered species, habitat fragmentation has resulted in small isolated populations with low genetic diversity and compromised adaptive potential. Although isolated patches of protected habitat can provide viable refuges for isolated or threatened genetic variation [9,72,73], these populations can face introgression from surrounding congeners upon secondary contact. To explore the genetic variation of the highly fragmented eastern wolf population in central Ontario, we collected genotype data for 304 canids using a reduced representation genotyping approach. This represents the first genome-wide SNP dataset with substantial sample sizes of representative populations. Our results show that eastern wolves, which are found in fragmented habitat patches across central Ontario [38,39], are genetically distinct due to the presence of alleles private to eastern wolves and carry a unique genetic composition of regional coyote and gray wolf alleles. Dense sampling surrounding the protected areas revealed a decreasing occurrence of eastern wolf genetic assignments with increasing distance from provincial parks. With few genetically identifiable eastern wolves found outside of provincial park boundaries, we detected signatures of interbreeding with coyotes or gray wolves predominantly outside of provincial parks, a result concordant with past genetic studies [37,38,39,74]. Adjacent to APP, we found admixture with coyotes in the south, and with gray wolves in the north. We detected eastern wolf alleles in admixed coyotes along the eastern Frontenac Axis (site 10), in admixed gray wolf populations northwest of KPP (sites 15–17, 19), and in canids of unknown taxonomy geographically intermediate of KPP and APP (sites 2 and 9). Collectively, our analyses reveal that eastern wolves are a geographically isolated yet distinct collection of genotypes, representing a unique genomic composition with their own ancestry not seen in other North American wolf populations, and are mostly restricted to small fragmented patches of protected habitat in central Ontario (e.g., APP, QEWPP, KPP, KHPP). As such, eastern wolves should be considered a priority for conservation.

While it is true that eastern wolves are mainly found in APP and other protected areas, previous work documents that resident, breeding eastern wolves are also patchily distributed in unprotected areas adjacent to APP [38]. The degree of genetic isolation of eastern wolves within Ontario’s protected areas, and the inference of these areas serving purely as sources for dispersers, calls into question their long-term sustainability. Although the decline of genetic diversity is not an immediate concern, isolation predicts that reduced effective size and potential inbreeding could increase the impact of genetic drift on degrading diversity. Furthermore, their low effective population size [39] makes them particularly susceptible to unexpected environmental changes. Eastern wolves are listed as threatened in Canada [42] and a 6340 km^2^ protective buffer zone comprising 39 geographic townships was established in 2001 around APP with further genetic surveys resulting in expanded protective zones [39].

Currently, human-caused mortality outside of protected areas is the biggest threat to eastern wolf conservation, with hunting and trapping banned within the buffer zone and in regions adjacent to Killarney, Queen Elizabeth II, and Kawartha Highlands Provincial Parks. Although they are listed as “threatened”, legal hunting and trapping continue outside the buffer zone. Much of the mortality stems from the inability to morphologically distinguish eastern coyotes from eastern wolves; consequently, eastern coyotes are included in the hunting and trapping ban in these buffer zones. Furthermore, assessment of historical and contemporary samples collected in APP concluded that historically intensive hunting facilitated coyote introgression, possibly due to reduction in availability of conspecific mates [74]. A similar hunting ban was implemented to promote the stability of the endangered red wolf (*C. rufus*) in North Carolina [75]. The goals of the ban were twofold: (1) reduce the mortality rate of an endangered species as the likelihood of red wolves being misidentified as coyotes was high, and (2) to maintain stable red wolf breeding pairs. Recreational hunting and trapping occur across Ontario and are suspected to facilitate coyote introgression into the eastern wolf genome [74], a dynamic also documented in red wolves [76,77]. Given the parallel threats to these wolves, eastern wolf conservation in Canada may serve as a model for red wolf recovery.

Conversely, admixture can result in beneficial genetic changes for a population if it produces new variation in fertile admixed offspring upon which adaptive selection can act (e.g., [78,79]). In this view, the hybridization that has led to the current eastern wolf population highlights an underappreciated mechanism that can facilitate adaptation through the recombination of genomes and phenotypes (e.g., [80,81]), which may be the foundation for the persistence of eastern wolves in a rapidly changing world. We want to encourage an innovative discussion regarding the quandary often faced in conservation policy: is the conservation priority to prevent introgression or should there be an acceptable level of admixture for eastern wolves and other endangered canids like red wolves? In the current climate of a changing world and shifts in species distributions [82], a plan for managed introgression would focus on preserving any eastern wolf genetic material in any genome regardless of their potential mosaic ancestry composition. Such an effort may prioritize and maintain individuals that carry admixed genomes as they are the source of greater genetic variation and potential adaptive capacity [79]. These efforts would greatly be supported by continued genomic and ecological monitoring of the source and admixed populations. The survival of even partial genomes underscores the need to focus conservation efforts on preserving diversity. Recently, such a phenomenon of persisting variation derived from extinct species was documented. Barlow and colleagues demonstrated that 0.9–2.4% of the brown bear (*Ursus arctos*) genome is derived from the extinct Late Pleistocene cave bear (*U. spelaeus*) species complex [83]. Their results are significant and suggest a reevaluation of species extinction, as their genetics remain active in admixed genomes.

The viewpoint described above prioritizes conserving genetic diversity across a gradient of admixed individuals. Adopting this view, canids of varying wolf and coyote ancestry should be conserved on the Ontario landscape to maintain unique eastern wolf genetic material and maximize evolutionary potential. A second viewpoint prioritizes ecological function and seeks to conserve roles that rare species play in ecosystems [84]. In the Ontario hybrid zone, wolves appear to be more consistent and predictable predators of large ungulate prey than coyotes or hybrids, which may serve to maintain stable predator-prey dynamics with moose and deer [85]. From this perspective, achieving a viable population of individuals with high eastern wolf ancestry, not simply the entire organism, is a priority in order to conserve their ecological role. The latter would also align with the legal mandate of the Canada and Ontario governments to attempt recovery of this threatened species via numerical and geographical expansion outside of APP. We suggest these seemingly disparate viewpoints are compatible from a management perspective. Scientists and managers agree that protecting eastern wolves also requires protection of coyotes and admixed canids given the difficulty of reliable identification. Thus, minimizing human-caused mortality of canids of varying wolf and coyote ancestry in areas adjacent to APP would seem to represent a unified strategy for (1) maximizing evolutionary potential, (2) conserving ecological function, and (3) attempting recovery of this threatened species on the Ontario landscape.

We find that eastern wolves represent a unique genomic cluster that is geographically isolated to patchy regions of central Ontario and possibly southern Quebec, with a general increase in admixture outside of protected areas. Their unique genetics provides a solid foundation to identify them as a conservation priority, especially given their low effective population size and degree of isolation. Further genomic assessment of canids in southern Quebec would help clarify the extent of the eastern wolf range outside of Ontario. Moving forward, conservation of the eastern wolf genome would benefit from the connection of current protective zones across the Ontario landscape.

## Figures and Tables

**Figure A1:**
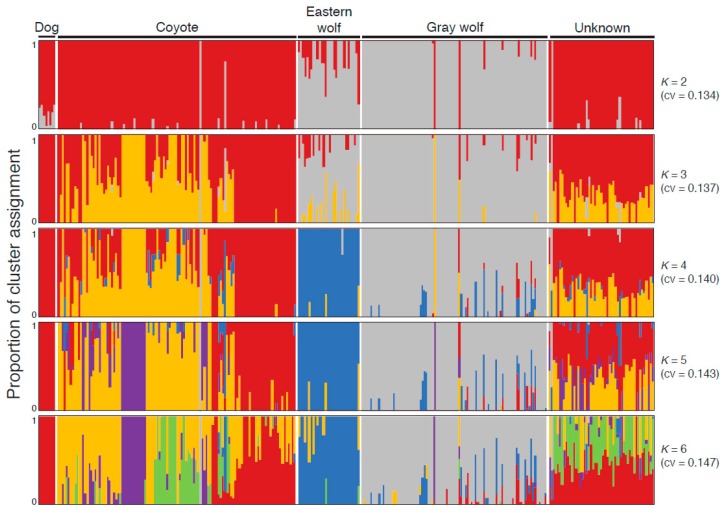
Genetic analysis of the 1157 neutral SNPs with a minor allele threshold of 1% and genotyped across 295 canids (n coyote = 116, eastern wolf = 30, gray wolf = 90, unknown canids = 51, and dogs = 8) using a maximum-likelihood method. The first six genetic partitions are displayed, each with their cross-validation (cv) values provided in parenthesis.

## Supplementary Materials

The following are available online at http://www.mdpi.com/2073-4425/9/12/606/s1, **Table S1:** Information for all samples including IDs, Species, Province/State, Location number for Figure 1, location abbreviation, specific location name if any, sample owner, latitude, and longitude, **Table S2:** Observed and expected heterozygosity (H_O_ and H_E_, respectively) across variant positions genotyped in 114 coyotes, 86 gray wolves, and 30 eastern wolves., **Table S3:** Private alleles estimated in each canid species across 5,665 SNPs genotyped across **A)** 253 canids from North America and **B)** 186 reference canids with high assignments at *K* = 3 to their respective clusters (*Q* > 0.9), **Table S4:** Individual Bayesian posterior probability (*Prob*) individual-level assignments at *K* = 3 for the 51 canids of unknown taxonomic affiliation from central Ontario as inferred from analysis of 3067 neutral SNPs and 186 reference canids (see Table S1)., **Table S5:** Likelihood values for migration event inferred from *TreeMix*, **Figure S1:** Allelic richness across 5665 SNPs genotyped in (**A**) 253 canids from North America (30 eastern wolves, 96 gray wolves, and 127 coyotes) and (**B**) 186 reference canids with high assignments at *K* = 3 to their respective clusters (*Q* > 0.9 n: coyote = 99, eastern wolf = 25, gray wolf = 62)., **Figure S2:** Box-and-whisker plot for the top 10 principal components (PC) representing 5665 SNP set genotyped in 281 canids (30 eastern wolves, 86 gray wolves, 114 coyotes, and 51 canids of unknown taxonomic affiliation from central Ontario)., **Figure S3:** Histogram of the differences in cross-validation (cv) values across 10 partitions from the analysis of genetic structure of 3067 neutral SNPs., **Figure S4:** The average *Q* value at *K*=3 per sampling site for the geographic region proximal to the provincial parks that contain eastern wolves (sites 1 and 2), **Figure S5:** Unrooted neighbor-joining cladogram based on pairwise genetic distances of 3,067 neutral SNPs showing clustering of 281 North American canids (species are indicated by branch colors, **Figure S6:** Details for the discriminant analysis of principal components (DAPC), **Figure S7:** Cluster assignments of 281 canids (30 eastern wolves, 86 gray wolves, 114 coyotes, and 51 canids of unknown taxonomic affiliation) from a discriminant analysis of principal components (DAPC) across 3,067 neutral SNPs., **Figure S8:** Tree topologies displaying from no admixture events (top left) to 10 migration events (lower right), indicated by colored arrows from 3,067 neutral SNP loci and 281 canids (n coyote = 114, eastern wolf = 30, gray wolf = 86, unknown canids = 51).
