# Supplementary material for: Population Genomic Analysis of North American Eastern Wolves (Canis lycaon) Supports Their Conservation Priority Status
Source: Genes (Basel). 2018 Dec 4;9(12):606. doi: 10.3390/genes9120606 (PMC6316216; doi:10.3390/genes9120606)

## Supplementary Materials

### Data Availability

All mapped BAM files are deposited on NCBI SRA (accession PRJNA490084). Meta-data can also be found in Table S1.

### Supplementary Tables and Figures

**Table S1.** Information for all samples including IDs, Species, Province/State, Location number for Figure 1, location abbreviation, specific location name if any, sample owner, latitude, and longitude. Associated results are also provided for each sample. Column *% missingness out of 16587 SNPs* provides a per-individual genotyping missingness, with bolded columns indicating samples that were excluded from all downstream analyses. Column *Assignment (eastern/gray/coyote)* provides the population proportions from *ADMIXTURE* using the 3,067 neutral SNP set. Column *Post-probability Reference* identifies which samples had $Q$>0.9 at $K$=3 and were used as reference individuals in a Bayesian posterior-probability assignment test. The *DAPC cluster* column contains the population assignment. Column *NJ tree* indicates broadly if the cluster membership of each sample is discordant with its taxonomic affiliation by listing the cluster to which it belonged. *TreeMix group* provides an identifier as to how samples were grouped for the *TreeMix* analysis. There are two sets of group columns for *Geneland* that provide a per-analysis population probability value for each individual in Figure 3. The last column of *% missingness out of 14248 SNPs +dogs* provides a per-sample genotyping missingness for the supplemental analysis that includes dogs, with bolded values to indicate which samples were excluded from analyses. (Abbreviations: APP, Algonquin Provincial Park; CLA, coyote; CLU, gray wolf; CLY, eastern wolf; SE, southeast; UNK, unknown)

**Table S2.** Observed and expected heterozygosity ($H_O$ and $H_E$, respectively) across variant positions genotyped in 114 coyotes, 86 gray wolves, and 30 eastern wolves. Pairwise *P*-values from 1-tailed *t*-tests of unequal variances are provided, with significant comparisons bolded ($P$<0.05).

| Species | $H_O$ | $H_E$ | Comparison to: | *P*-values ($H_O$, $H_E$) |
|---|---|---|---|---|
| *5K SNPs* | | | | |
| Coyote | 0.061 | 0.074 | Gray wolf | **$8.89 \times 10^{-8}$, $8.46 \times 10^{-5}$** |
| | | | Eastern wolf | **0.00312**, 0.18 |
| Gray wolf | 0.069 | 0.080 | Eastern wolf | **0.0302, $2.80 \times 10^{-5}$** |
| Eastern wolf | 0.065 | 0.072 | -- | |
| *Neutral 3K SNPs* | | | | |
| Coyote | 0.052 | 0.056 | Gray wolf | 0.0722, 0.397 |
| | | | Eastern wolf | 0.383, **0.00848** |
| Gray wolf | 0.055 | 0.056 | Eastern wolf | 0.0754, **0.00786** |
| Eastern wolf | 0.052 | 0.052 | -- | -- |

**Table S3.** Private alleles estimated in each canid species across 5,665 SNPs genotyped across **A)** 253 canids from North America and **B)** 186 reference canids with high assignments at $K$=3 to their respective clusters ($Q$>0.9) (see Table S1). (Abbreviations: n, sample size; s.e., standard error; var, variance)

**A)**

| Species (n) | Number of Private alleles | Allelic richness (s.e., var) |
|---|---|---|
| Coyote (127) | 1157 | 1.65 (0.12, 0.00) |
| Eastern wolf (30) | 12 | 1.49 (0.22, 0.01) |
| Gray wolf (96) | 395 | 1.54 (0.17, 0.01) |

**B)**

| Species (n) | Number of Private alleles | Allelic richness (s.e., var) |
|---|---|---|
| Coyote (99) | 1718 | 1.59 (0.13, 0.00) |
| Eastern wolf (25) | 62 | 1.43 (0.22, 0.01) |
| Gray wolf (62) | 574 | 1.45 (0.20, 0.01) |

**Table S4.** Individual Bayesian posterior probability (*Prob*) individual-level assignments at *K*=3 for the 51 canids of unknown taxonomic affiliation from central Ontario as inferred from analysis of 3,067 neutral SNPs and 186 reference canids (see Table S1). Highest posterior probabilities are bolded.

| Location ID* | Sample ID | *Prob*(Coyote) | *Prob*(Eastern wolf) | *Prob*(Gray wolf) |
|---|---|---|---|---|
| 1 | 8655 | **0.916** | 0.054 | 0.030 |
| 1 | 8656 | **0.964** | 0.025 | 0.011 |
| 1 | 8659 | **0.892** | 0.067 | 0.041 |
| 1 | 8661 | **0.964** | 0.030 | 0.006 |
| 1 | 8662 | **0.890** | 0.071 | 0.039 |
| 1 | 8663 | **0.911** | 0.049 | 0.041 |
| 1 | 8664 | **0.929** | 0.044 | 0.027 |
| 2 | 8645 | **0.901** | 0.069 | 0.030 |
| 2 | 8647 | **0.906** | 0.065 | 0.030 |
| 3 | 8654 | **0.909** | 0.059 | 0.031 |
| 4 | 8605 | **0.927** | 0.063 | 0.010 |
| 4 | 8607 | **0.941** | 0.037 | 0.021 |
| 4 | 8608 | **0.883** | 0.057 | 0.060 |
| 5 | 8606 | **0.867** | 0.092 | 0.041 |
| 6 | 8649 | **0.925** | 0.061 | 0.014 |
| 6 | 8650 | **0.934** | 0.048 | 0.019 |
| 6 | 8651 | **0.942** | 0.044 | 0.014 |
| 6 | 8652 | **0.936** | 0.043 | 0.021 |
| 7 | 8623 | **0.896** | 0.070 | 0.034 |
| 7 | 8624 | **0.938** | 0.034 | 0.028 |
| 7 | 8626 | **0.940** | 0.031 | 0.030 |
| 8 | 8658 | **0.908** | 0.063 | 0.029 |
| 8 | 8660 | **0.924** | 0.051 | 0.024 |
| 8 | 8674 | **0.913** | 0.060 | 0.027 |
| 9 | 8628 | **0.905** | 0.065 | 0.030 |
| 9 | 8629 | **0.917** | 0.047 | 0.036 |
| 9 | 8630 | **0.886** | 0.056 | 0.058 |
| 10 | 8603 | **0.872** | 0.035 | 0.093 |
| 10 | 8604 | **0.841** | 0.082 | 0.077 |
| 11 | 8644 | **0.923** | 0.056 | 0.021 |
| 12 | 8610 | **0.852** | 0.089 | 0.060 |
| 12 | 8611 | **0.913** | 0.035 | 0.052 |
| 12 | 8613 | **0.941** | 0.034 | 0.025 |
| 12 | 8614 | **0.924** | 0.045 | 0.031 |
| 12 | 8616 | **0.932** | 0.044 | 0.024 |

| | | | | |
|---|---|---|---|---|
| 12 | 8618 | **0.873** | 0.079 | 0.048 |
| 12 | 8620 | **0.931** | 0.053 | 0.016 |
| 12 | 8621 | **0.885** | 0.061 | 0.054 |
| 13 | 8634 | **0.929** | 0.051 | 0.019 |
| 13 | 8635 | **0.923** | 0.057 | 0.020 |
| 13 | 8636 | **0.919** | 0.059 | 0.022 |
| 13 | 8637 | **0.917** | 0.063 | 0.020 |
| 13 | 8638 | **0.925** | 0.051 | 0.024 |
| 13 | 8639 | **0.906** | 0.070 | 0.025 |
| 13 | 8640 | **0.931** | 0.042 | 0.027 |
| 13 | 8641 | **0.868** | 0.090 | 0.041 |
| 13 | 8642 | **0.900** | 0.074 | 0.026 |
| 13 | 8643 | **0.932** | 0.054 | 0.014 |
| 13 | 8648 | **0.935** | 0.030 | 0.035 |
| 14 | 8633 | **0.934** | 0.026 | 0.040 |
| 15 | 8632 | **0.905** | 0.065 | 0.030 |

*****The location ID details are described in Fig. 1 and Table S1.

**Table S5.** Likelihood values for migration event inferred from *TreeMix*. The starting ln(likelihood) value for $m$=0 is 233.31. (Abbreviations: $m$, number of migration events)

| $m$ | Exiting ln(likelihood) |
|---|---|
| 1 | 253.26 |
| 2 | 266.73 |
| 3 | 273.51 |
| 4 | 293.54 |
| 5 | 300.96 |
| 6 | 299.89 |
| 7 | 300.29 |
| 8 | 302.59 |
| 9 | 302.17 |
| 10 | 302.38 |

**Figure S1.** Allelic richness across 5,665 SNPs genotyped in **A)** 253 canids from North America (30 eastern wolves, 96 gray wolves, and 127 coyotes) and **B)** 186 reference canids with high assignments at *K*=3 to their respective clusters (*Q*>0.9 n: coyote=99, eastern wolf=25, gray wolf=62).

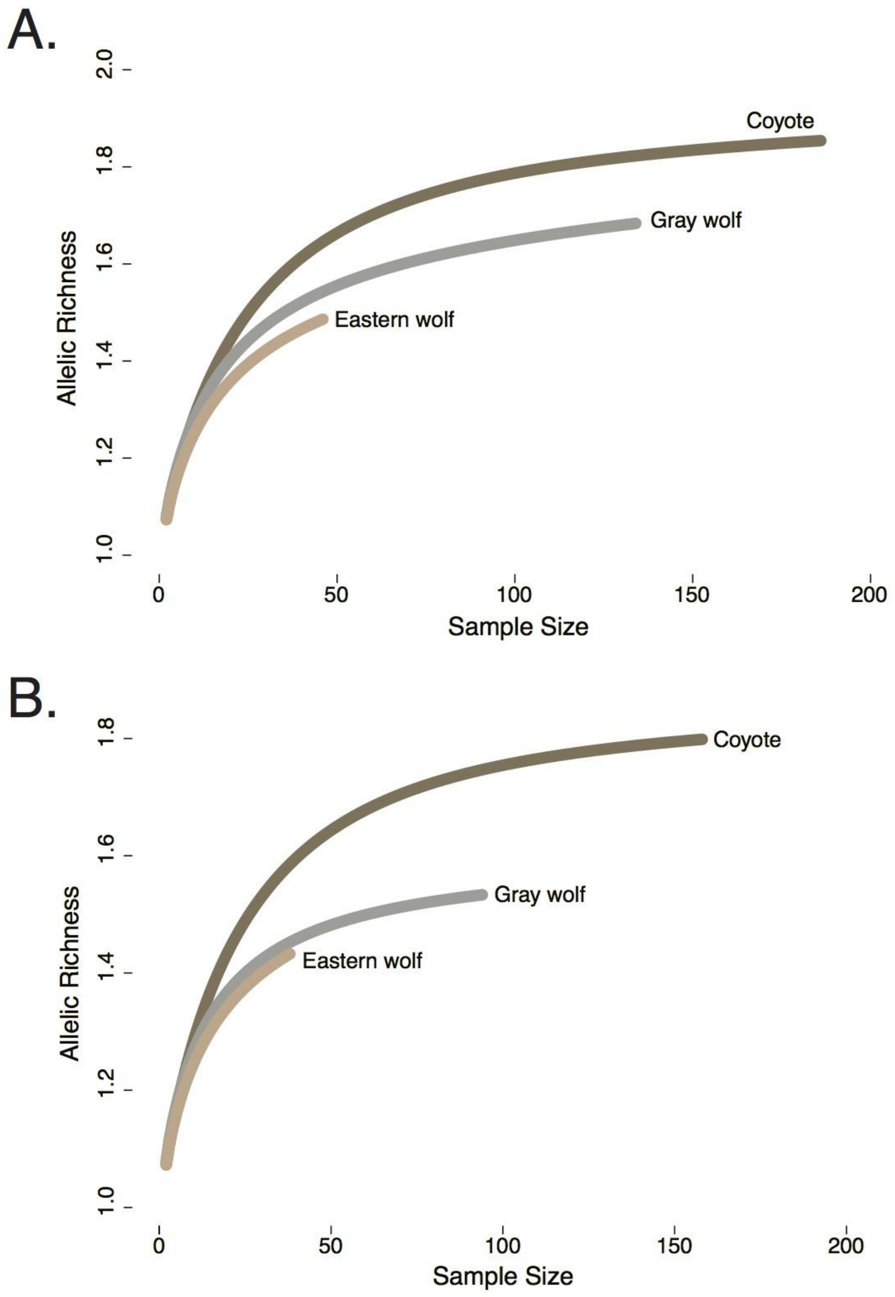

**Figure S2.** Box-and-whisker plot for the top 10 principal components (PC) representing 5,665 SNP set genotyped in 281 canids (30 eastern wolves, 86 gray wolves, 114 coyotes, and 51 canids of unknown taxonomic affiliation from central Ontario). Percent variation for each PC is provided along the Y-axis.

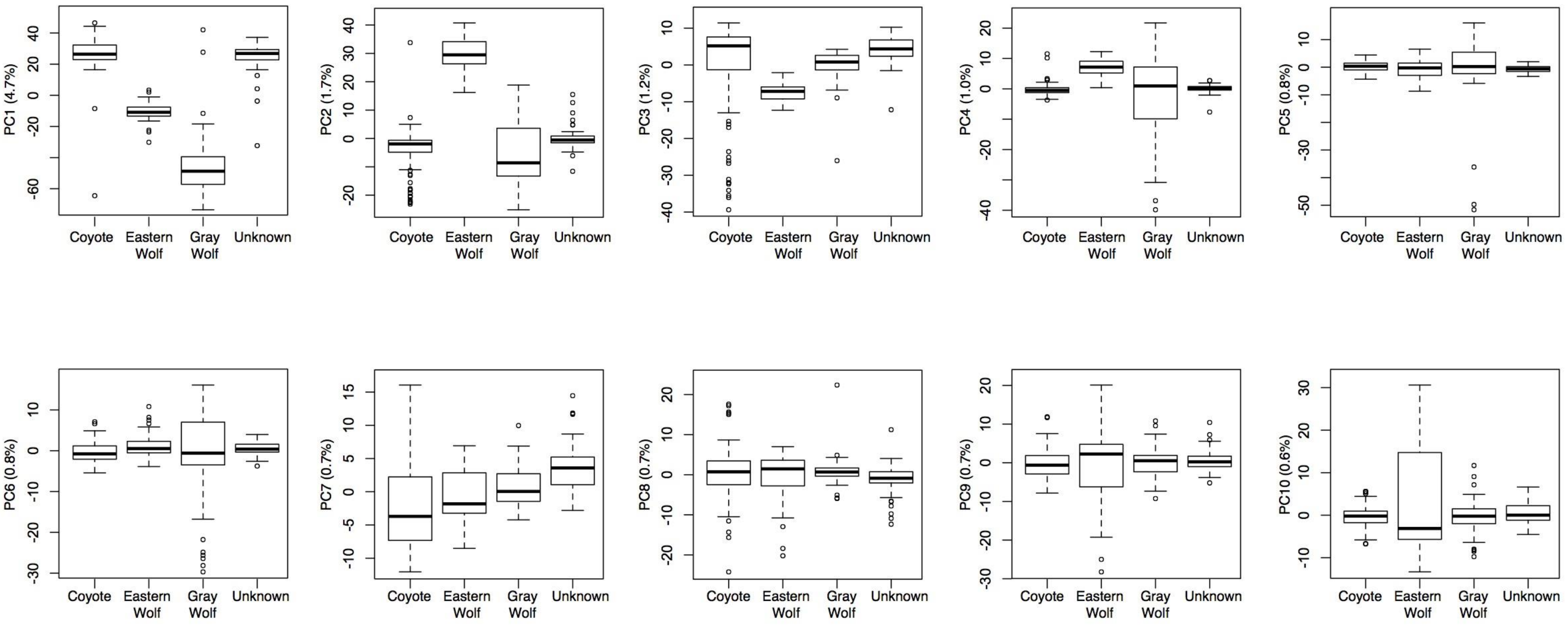

**Figure S3.** Histogram of the differences in cross-validation (*cv*) values across 10 partitions from the analysis of genetic structure of 3,067 neutral SNPs.

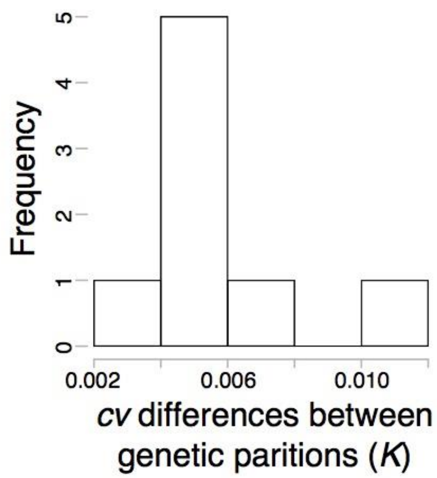

**Figure S4.** The average *Q* value at *K*=3 per sampling site for the geographic region proximal to the provincial parks that contain eastern wolves (sites 1 and 2). Pie charts from either coyote or gray wolf sites are labeled as “cla” or “clu”, respectively, and numbered following from Figure 1. Pie charts labeled as “unk” represent the sites of canids lacking a known taxonomic classification. This figure is a zoomed-in version of Figure 2C.

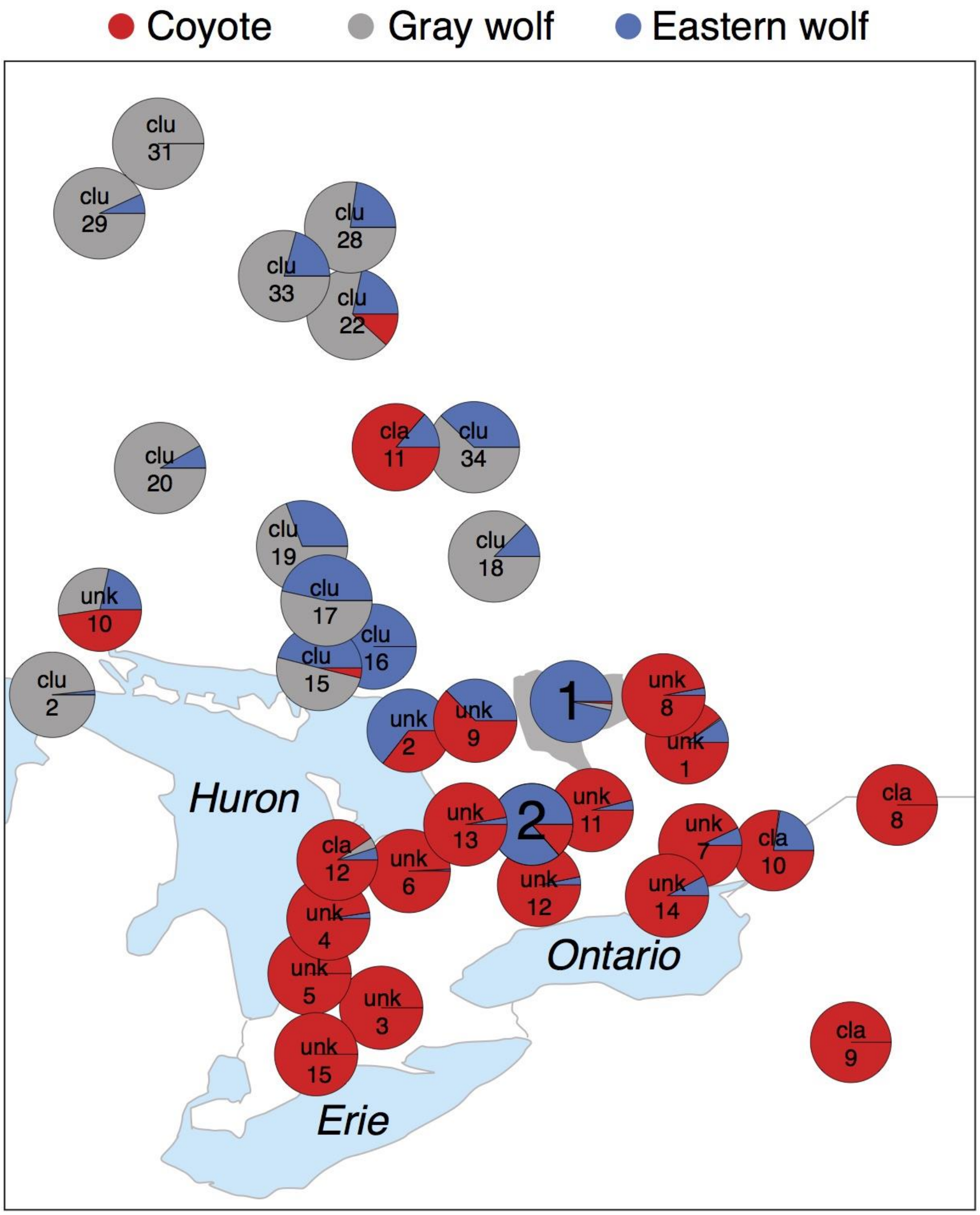

**Figure S5.** Unrooted neighbor-joining cladogram based on pairwise genetic distances of 3,067 neutral SNPs showing clustering of 281 North American canids (species are indicated by branch colors). Black and red dots represent coyotes, eastern wolves, and gray wolves that had $Q<0.9$ to their respective species in *ADMIXTURE* at $K=3$ and $K=6$, respectively.

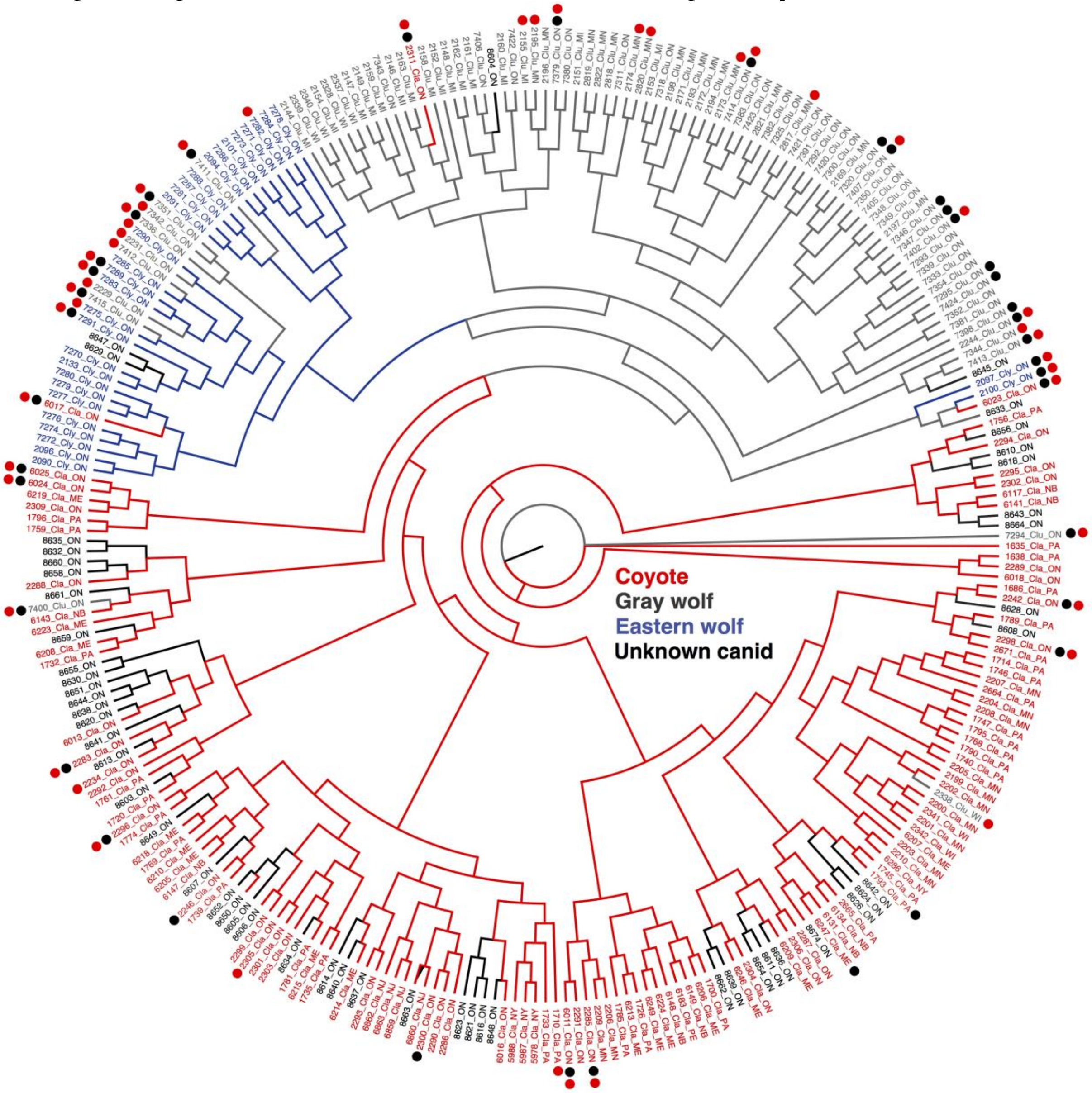

**Figure S6.** Details for the discriminant analysis of principal components (DAPC). **A)** Finding the number of PCs to retain. **B)** Bayesian Information Criterion to identify the best fit number of clusters. **C)** Discriminant analysis to identify the number of PCs to retain. **D)** Analysis of eigenvalues for each discriminant function.

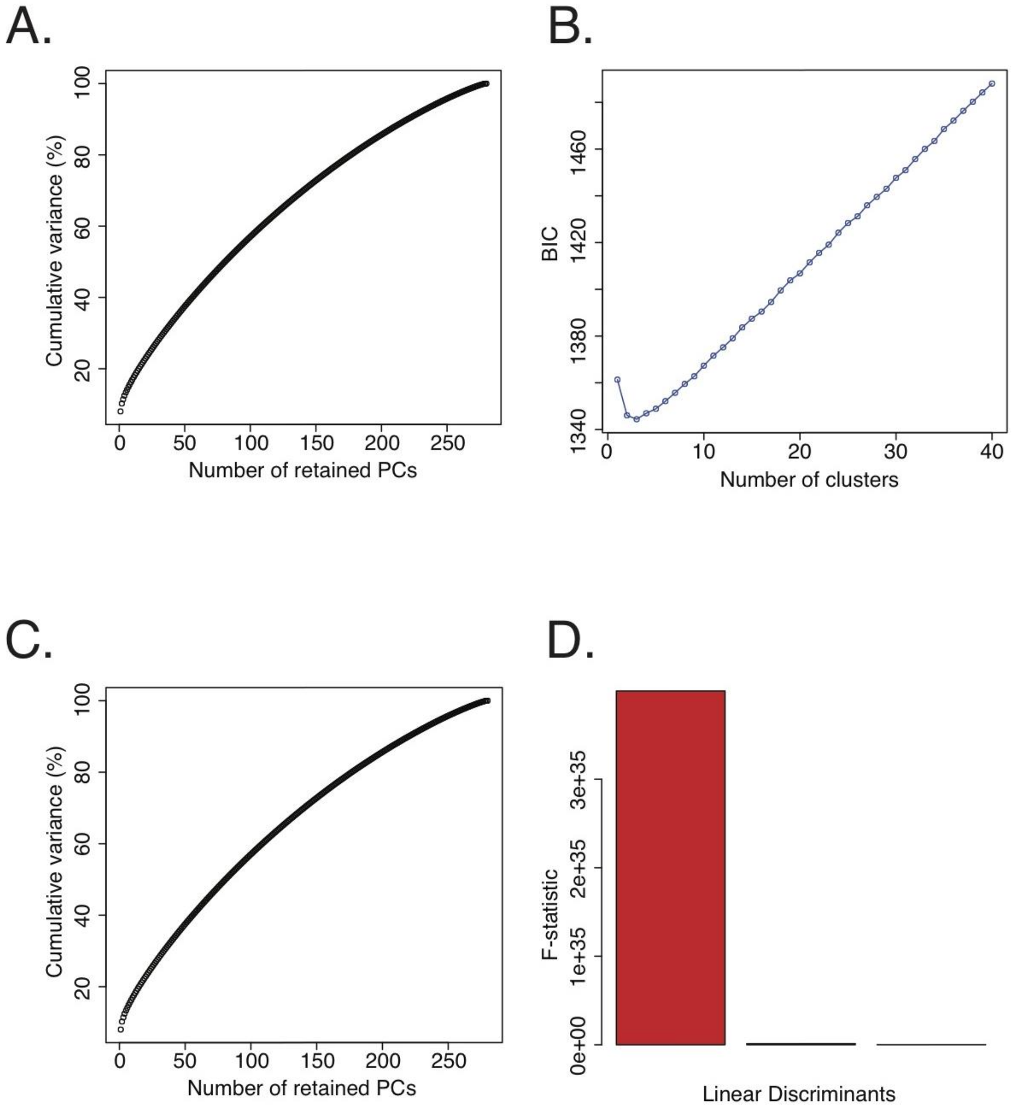

**Figure S7.** Cluster assignments of 281 canids (30 eastern wolves, 86 gray wolves, 114 coyotes, and 51 canids of unknown taxonomic affiliation) from a discriminant analysis of principal components (DAPC) across 3,067 neutral SNPs.

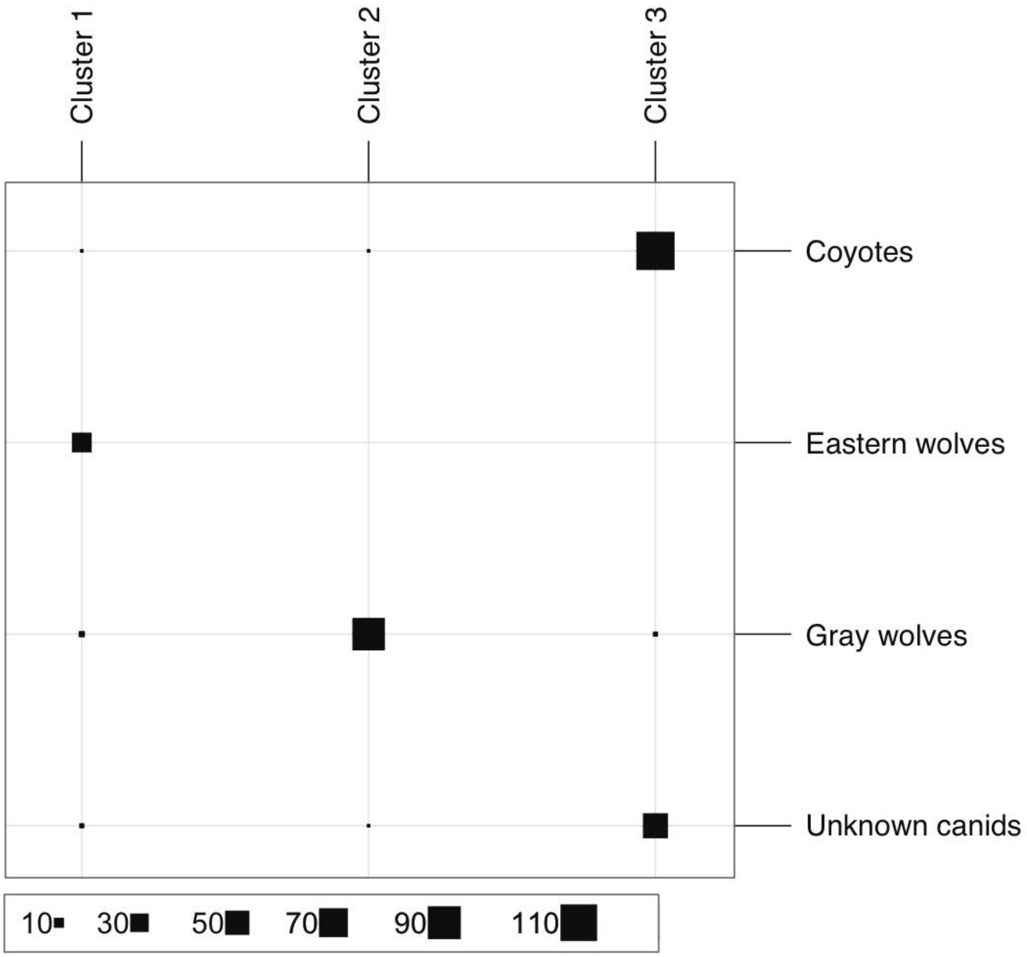

**Figure S8.** Tree topologies displaying from no admixture events (top left) to 10 migration events (lower right), indicated by colored arrows from 3,067 neutral SNP loci and 281 canids (n coyote=114, eastern wolf=30, gray wolf=86, unknown canids=51). The color bar indicates the migration weight, reflecting the contribution of the parental population(s). The X-axis indicates the drift parameter, which often approximates the impact of effect size. Node labels are formatted as a three-letter species code (cla, *C. latrans*; cly, *C. lycaon*; clu, *C. lupus*) followed by their sample location as identified in Table S1 and Figure 1.

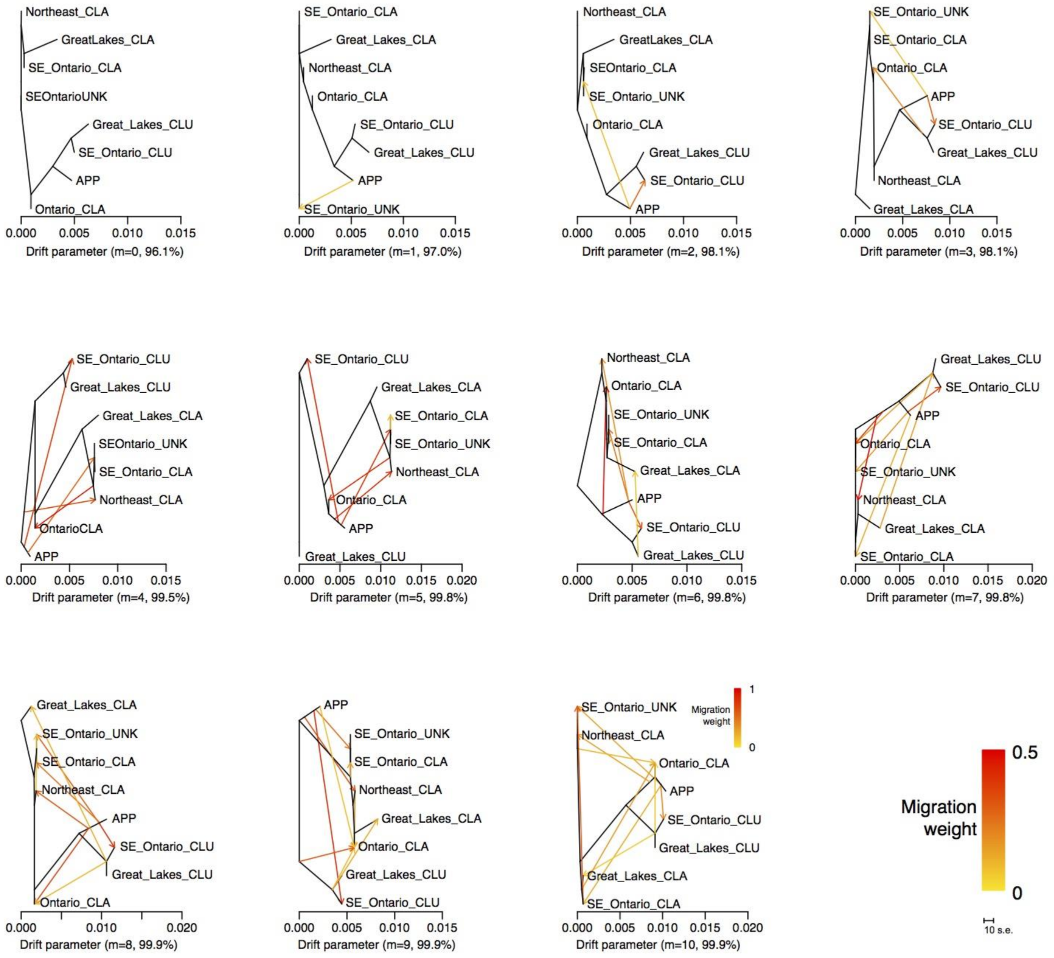

**Supplemental Note: A brief summary of the analysis that included 13 domestic dogs.**

This note describes the methods and results for a separate dataset of 317 canids, which is an expansion of the 304 wild canids described in the main text to include 13 domestic pet-owned dogs collected from southeastern Ontario (see Table S1). All of the dogs used in this study were from previous work [1]. Briefly, samples were collected between 2006 and 2011 during routine blood sampling by veterinarians at a local pet clinic in Peterborough, Ontario. Dogs are from mixed breeds of approximately 25-30 kg in weight [1]. These samples were prepared for RADseq and reads processed as described in the main text. All the software used are as described in the main text. We obtained genotypes for 14,248 SNPs (referred to as 14K SNP set) discovered across 127 coyotes, 96 gray wolves, 30 eastern wolves, 51 canids of unknown taxonomic assignment, and 13 domestic dogs. We excluded 22 individuals with a significantly high level of missingness (i.e. $\geq 93^{rd}$ percentile of the missingness distribution or missingness$\geq$0.28): 11 coyotes (Pennsylvania n=10, Ontario n=1), six gray wolves (Michigan n=2, Ontario n=4), and five dogs (Table S1). These 22 individuals were removed from all subsequent analyses of the remaining 295 canids (coyote n=116, eastern wolf n=30, gray wolf n=90, unknown canids n=51, and dogs n=8). From this final set of samples, we obtained a statistically unlinked set of 7,810 SNP loci after filtering for linkage disequilibrium (LD) in *PLINK* using the flag and parameters --indep-pairwise 50 5 0.5 and retaining SNPs in Hardy-Weinberg Equilibrium (HWE) in *PLINK* (--hardy; $P$>0.05). This SNP set will be hereafter referred to as the “neutral SNP set”. SNPs were then filtered to retain sites with a minor allele frequency of 1%, resulting in 1,157 neutral SNPs.

We evaluated genetic structure using *ADMIXTURE* with details as described in the main text and found that dogs are a unique, discrete genetic cluster that does not explain the genetic clustering of eastern wolves of APP, with inconsequential amounts of dog cluster assignments for eastern wolves (Fig. A1). Although two genetic clusters were the most supported, there are insignificant differences in fit between $K$=2-6 genetic partitions. We thus encourage caution when selecting the most supported level of genomic partitioning, with each influenced by demography, data filtering, and sample size.

References

1. Wheeldon, T.J., Rutledge, L.Y., Patterson, B.R., White, B.N., Wilson, P.J. Y-chromosome evidence supports asymmetric dog introgression into eastern coyotes. *Ecol Evol* **2013** *3*(9), 3005-3020.

**Figure A1.** Genetic analysis of the 1,157 neutral SNPs with a minor allele threshold of 1% and genotyped across 295 canids (n coyote=116, eastern wolf=30, gray wolf=90, unknown canids=51, and dogs=8) using a maximum-likelihood method. The first six genetic partitions are displayed, each with their cross-validation (*cv*) values provided in parenthesis.

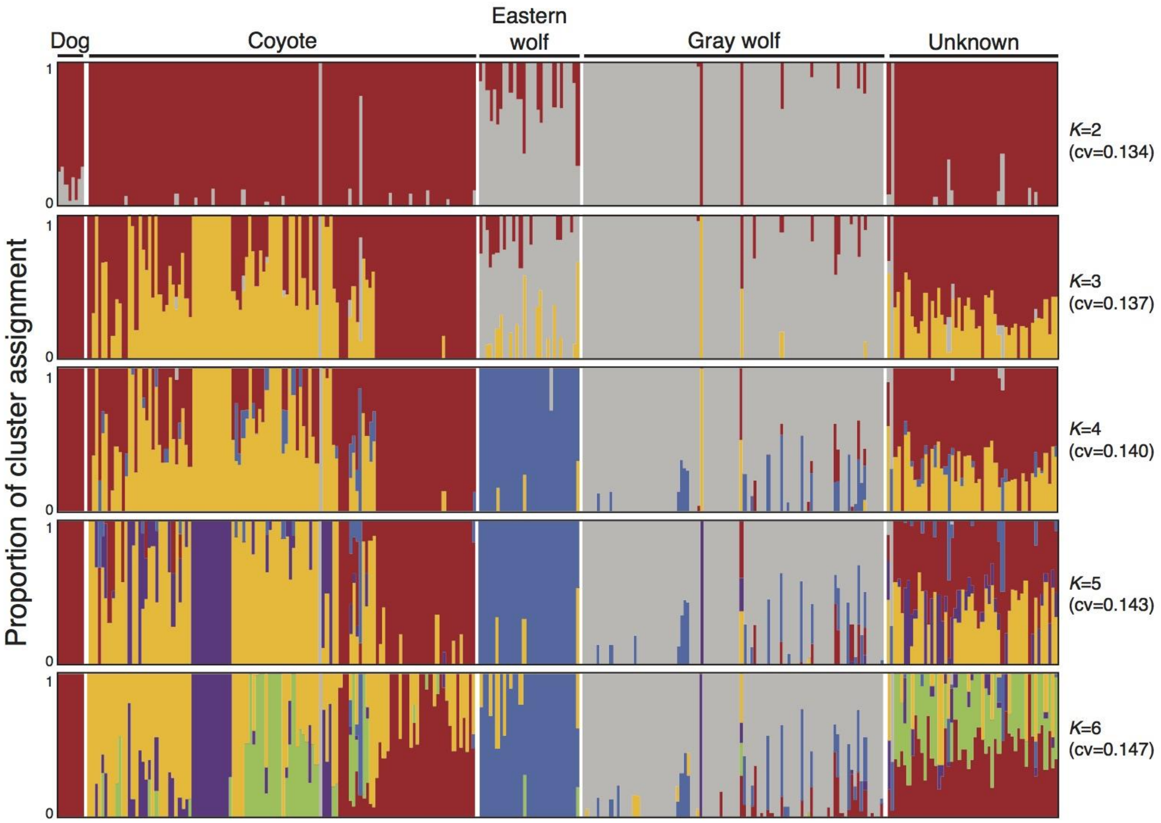

Supplement: Supplementary file 1 [file genes-09-00606-s001.zip › genes-379820-supplementary-final.pdf]
